# A narrative review on the role of cognition, nutrition and energy availability in athletes of competitive sports to combat RED-S

**DOI:** 10.7717/peerj.18849

**Published:** 2025-01-31

**Authors:** Subalatha M., Dhanush Rachaveti, Amutha S., Ponpandi M.

**Affiliations:** 1 R&D, Heatechs Instruments, Chennai, Tamilnadu, India; 2 School of Electronics Engineering, Vellore Institute of Technology Chennai, Tamil Nadu, India; 3 School of Computer Science and Engineering, Vellore Institute of Technology Chennai, Tamil Nadu, India; 4 Department of Physical Education, Vellore Institute of Technology Chennai, Tamil Nadu, India

**Keywords:** Cognition in athletes, Nutrition in athletes, Energy deficiency in athletes, RED-S, Low energy availability in athletes

## Abstract

**Background:**

In the present scenario, competitive sports require athletes to achieve a phenomenal balance between cognitive abilities, motor skills, nutritional intake, and energy deficiencies. Such stability would enable the athletes to excel in their sporting field. Evidence shows that athletes develop specific cognitive abilities based on their sporting field. Nutrition is vital in creating an athlete’s cognitive ability and physical needs required to participate in competitive sports. The reduction in the intake of nutrients required before, after and during sports participation could result in relative energy deficiency in sports (RED-S), affecting the parts of the body.

**Methods:**

The rationale behind the survey is to understand the role of nutrition and energy deficiency on the athletes’ cognitive abilities. The review’s research areas were identified as athletes’ cognition and nutrition in the context of RED-S. Search keywords were found based on the research area, such as “cognitive”, “nutrition”, and “energy deficiency/availability” in athletes. The search keywords were combined to form search queries (SQs). SQs were used to carry out the search on the Web of Science and Scopus databases.

**Results:**

Sports play an important role in athletes’ cognitive abilities, such as decision-making, attention, memory, *etc*. Nutritional intakes, such as caffeinated, carbohydrate, alkaline, and protein-based supplements and diets, also significantly affect athletes’ cognitive and motor abilities. Low energy availability (LEA) causes cognitive and physical health problems in both female and male athletes.

**Conclusion:**

The review identified that nutrition and LEA play crucial roles in athletes’ cognitive performance. Deficits in nutritional intake and energy availability lead to RED-S. Hence, cognitive performance could be used as an early indication to identify the nutritional and energy deficits in advance, enabling athletes to combat RED-S.

## Introduction

Cognitive abilities play an essential role in the performance of physical activities (McMorris, 2016; Warburton & Bredin, 2017). In sports, elite athletes strenuously participate in physical activities to show their remarkable performances (Kramer & Erickson, 2007; Etnier & Chang, 2009). Certain studies have shown that highly cognitively demanding physical tasks have more substantial and positive effects on athletes’ cognitive abilities (Diamond & Ling, 2016, 2019; Gu et al., 2019). Studies have also documented that the developed cognitive skills are connected with anatomical and physiological changes in the brain (Chaddock et al., 2011; Voelcker-Rehage & Niemann, 2013). Certain studies have hypothesized that functional and anatomical changes in the brain could be attributed to the possible activation of physical activity-sensitive neurotrophins. These physical activity-sensitive neurotrophins are vascular endothelial growth factor (VEGF), insulin-like growth factor-1 (IGF-1) and brain-derived neurotrophic factor (BDNP) (Carro et al., 2001; Cotman & Berchtold, 2002; Licht et al., 2011). Any competitive sport combines several specific physical activities to accomplish a sports goal. Therefore, participation in such sports could activate these physical activity-sensitive neurotrophins, promoting cognitive abilities for athletes in particular sports.

Nutrition for athletes is quintessential, and appropriate nutrients provide athletes with sufficient energy to maintain the cognitive and motor functions required to perform specific physical activity in a given sport (Tardy et al., 2020; Amawi et al., 2024). The main categories of nutrients for athletes include carbohydrates (glucose), proteins (amino acids (oily fish)), lipids (omega 3 fatty acids), micronutrients (zinc (whole grains), iodine (eggs, chicken), B vitamins (soya beans, oats)), and hydration (water) (Karpecka & Fraczek, 2022). Further, correct nutrition intake will produce appropriate energy to balance the optimal body function and sports performance. In recent times, athletes with proper physical and cognitive abilities have collapsed suddenly due to cardiac arrest (Guillen & Watkins, 2024). One of the critical reasons for such collapse is unnoticed energy deficiency or low energy availability (LEA) due to improper intake of nutrients. The energy deficiency caused relative to balance between energy intake and expenditure required for the body’s basal functions such as homeostasis, growth, daily living and physical activities related to a specific sport is called relative energy deficiency in sports (RED-S) (Mountjoy et al., 2018). RED-S could result in osteoporosis, premature cardiovascular failure, and menstrual cycle dysfunction in athletes (Guillen & Watkins, 2024).

Cognitive functions such as decision-making, inhibitory control, cognitive flexibility, working memory, and information processing speed have been vital to achieving appropriate motor performance to achieve sports goals (Di Russo et al., 2010; Wang et al., 2017; Yu et al., 2017; Holfelder et al., 2020). These cognitive and motor functions tend to depend on the ingestion of the appropriate nutrients for the athletes (Rodriguez-Giustiniani et al., 2019; Pirmohammadi et al., 2023; Mabrey et al., 2024). Cognitive restraint is defined as a conscious restriction to consume lesser nutrients in fear of becoming overweight or losing weight (Jurov et al., 2021b). Cognitive restraint could be one of the possible factors for LEA in RED-S (Jurov et al., 2021b). Such deliberate irregular nutrient intake affects the cognitive and motor performance of the athletes, giving the relationship between RED-S and cognitive performance. Even though separate reviews are available on cognition, nutrition and energy deficiencies for athletes, a collective attempt to highlight the dependencies of these facets remains a gap in the literature (Silva, Conte & Clemente, 2020; Tardy et al., 2020; Furley, Schütz & Wood, 2023; Melin et al., 2024; Amawi et al., 2024). The rationale behind the present review is to gather scientific evidence on the dependencies of cognitive abilities, nutritional intake and energy deficiencies among athletes involved in competitive sports. Cognition, nutrition and energy deficiencies play vital roles in addressing the challenges faced by athletes, both physically and mentally. The current review aims to investigate the following questions:

1. Impact of cognitive functions, such as decision-making, disinhibition, memory, and attention, on the motor performance of athletes in competitive sports.

2. The role of reduced nutritional intake and LEA on the cognitive and motor abilities of the athletes.

3. Highlight the possibilities of using cognitive performance as first-level indicators to identify nutritional and energy deficits in athletes to prevent RED-S/female athlete triad (FAT). FAT was previously used to represent the complications associated with RED-S. FAT was defined as the combination of eating disorders and irregular menstrual cycles, finally leading to low oestrogen hormones and low bone mineral density (Mountjoy et al., 2018). FAT was replaced by a term called RED-S, which also accounts for conditions seen as prevalent among male athletes (Mountjoy et al., 2018).

## Methodology

The research area of the literature survey is the cognition, nutrition and energy availability/deficiency of athletes. Based on the research area, essential search keywords were identified as “cognitive”, “nutrition”, and “energy deficiency/availability” in athletes. The search for research articles was carried out on the Web of Science and Scopus databases. Research articles that were available based on the search were taken into consideration. The articles as early as 1978 till the present date were considered for further analysis (Ferraz et al., 2024).

### Search queries

The identified search keywords were used to form search queries (SQs) to search the databases for research articles. The syntax for SQs was different for different databases. They were usually connected with a connecting operator (AND/OR) and a search tag indicating a specific search keyword in a research article. The research article with experimental study designs was considered. Hence, the keyword “study design” and the primary search keywords were used to form the SQs.

The SQs were used in the following format and varied between the databases. For example, SQs for the Web of Science database are mentioned below,

SQ1: ALL = (athletes) AND ALL = (cognitive) AND ALL = (study design).

SQ2: ALL = (athletes) AND ALL = (nutrition) AND ALL = (study design).

SQ3: ALL = (athletes) AND (ALL = (energy availability) or ALL = (energy deficiency)) AND ALL = (study design).

SQ4: ALL = (athletes) AND (ALL = (energy availability) or ALL = (energy deficiency)) AND ALL = (study design) AND ALL = (cognitive).

SQ5: ALL = (athletes) AND (ALL = (energy availability) or ALL = (energy deficiency)) AND ALL = (study design) AND ALL = (nutrition).

SQ6: ALL = (athletes) AND ALL = (nutrition) AND ALL = (study design) AND ALL = (cognitive).

SQ7: ALL = (athletes) AND ALL = (cognitive) AND ALL = (study design) AND ALL = (nutrition) AND (ALL = (energy availability) or ALL = (energy deficiency));

### Selection criteria

The articles were further scrutinised using the inclusion and exclusion criteria. The research articles were included only if they satisfied the following conditions: 1. Articles relevant to cognition, nutrition and energy availability/deficiency for athletes were only considered. 2. Original research articles with experimental data as per point 1 were considered. 3. Competitive sports such as soccer, volleyball, tennis, badminton, taekwondo, boxing, *etc*., that require the use of cognitive functions such as decision-making, disinhibition, working memory, and cognitive flexibility to accomplish the goal of the sport were considered. 4. Original research articles with high reporting standards as per Consolidated Standards of Reporting Trials (CONSORT) were considered (Cuschieri, 2019). The research articles were excluded if they were article types such as reviews, meta-analysis, conference abstracts, and books. The articles with injuries imposed by causes other than RED-S syndrome, such as impact-based concussion, *etc*. were excluded from the survey. The research articles on elderly retired athletes, wheelchair-based athletes, amputees, and non-athletes were also excluded. The studies conducted on simulated avatars using Virtual Reality and Augmented Reality and those studies that did not focus on investigation questions were excluded.

### Quality assessment

The research articles that were included in the review were further assessed for quality. The present review methodology adopted the high standards of reporting provided by CONSORT and Delphi’s list for quality assessment (Verhagen et al., 1998; Cuschieri, 2019; Sirohi et al., 2022). The parameters provided by the CONSORT and Delphi lists remain suitable for experimental study designs. Hence, the adopted parameters assessed the research articles *n* = 39 included in the current review. The following CONSORT and Delphi list parameters were considered for quality assessment. They are

1) Experimental design (ED): The study should address the problem statement with clear objectives (OB). It should also have a clear and detailed report on the experimental design, including the trial design (TD), randomization (R), participant details (P), interventions (I), outcomes (O), sufficient sample size (SS), clarity in study type (ST).
2) Statistical methods (SM): The study should have segregated the population based on the study type for within-group (W) and between-group (B) comparisons.
3) Interpretation of results with statistics (IS): The study should appropriately represent the outcomes mentioned in the experimental design section. The outcome of the research study will be determined with appropriate statistical interpretations such as mean, standard deviations, standard error of means, confidence intervals, *P* values, significant levels, effect sizes, *etc*.
4) The quality assessment showed that the articles considered for the study were rated on the following scale of quality: 1–4: Low quality (L); 5–7: Medium quality (M); 8–10: High quality (H).
5) The quality assessment showed that review articles considered for the study had a quality assessment of H = 26, M = 13, and L = 0. The quality assessment table highlights that around ~67% of articles considered were of high quality, and around ~33% were of medium quality. The quality assessment analysis is shown in Table 1.

**Table 1:** The quality assessment performed on the list of review articles.

Research articles	Objectives (OB)	Trial design (TD)	Participant details (PD)	Randomization (R)	Interventions (I)	Outcomes (O)	Sample size (SS)	Study type (ST)	Statistical methods (SM)	Interpretation of results with statistics (IS)	Total	Quality assessment grade
Staiano et al. (2022)	1	0	1	0	1	1	1	1	0	1	7	M
Friebe et al. (2024)	1	0	1	1	1	1	1	1	1	1	9	H
Gantois et al. (2020)	1	1	0	1	1	1	1	1	1	1	9	H
Piggott et al. (2019)	1	1	1	0	1	1	1	1	1	0	8	H
Lucia, Bianco & Di Russo (2023)	1	1	1	1	0	1	1	1	1	1	9	H
Zhu et al. (2022)	1	1	1	1	0	1	1	1	1	1	9	H
Huang et al. (2024)	1	1	1	0	0	1	0	1	1	1	7	M
Song, Xiang & Zhong (2024)	1	1	1	0	0	1	1	1	1	1	8	H
Yongtawee et al. (2022)	1	1	1	1	0	1	1	1	1	1	9	H
Koch & Krenn (2021)	1	1	1	1	0	1	1	0	1	1	8	H
Zhang et al. (2022)	1	1	1	0	0	1	1	1	1	1	8	H
Zhang et al. (2021)	1	1	1	0	0	1	1	1	1	1	8	H
Zheng et al. (2024)	1	1	1	1	0	1	0	1	1	1	8	H
Buzdagli et al. (2024)	1	1	1	0	0	1	1	1	1	1	8	H
Philpott et al. (2018)	1	1	0	0	1	1	1	0	1	1	7	M
Pirmohammadi et al. (2023)	1	0	1	1	1	1	0	1	1	1	8	H
Kawamura, Nemoto & Sugita (2023)	1	1	1	1	1	1	1	1	1	1	10	H
Stankiewicz et al. (2021)	1	1	1	1	1	1	0	1	0	1	8	H
Limmer, Eibl & Platen (2018)	1	1	1	1	1	1	0	1	1	1	9	H
Rodriguez-Giustiniani et al. (2019)	1	1	1	1	0	1	0	1	1	1	8	H
Ho et al. (2018)	1	1	1	1	0	1	0	1	1	1	8	H
Egesoy & Oksuzoglu (2020)	1	1	1	1	0	1	1	0	1	1	8	H
Durkalec-Michalski et al. (2020)	1	1	1	1	0	1	0	1	1	1	8	H
Mabrey et al. (2024)	1	1	1	1	1	1	0	1	1	1	9	H
Heileson et al. (2021)	1	1	1	0	0	1	1	0	1	1	7	M
Sun, Cooper & Chak-Fung Tse (2020)	1	1	1	0	1	1	0	1	1	1	8	H
Zhu et al. (2020)	1	1	1	1	1	1	0	1	1	1	9	H
Goulart et al. (2023)	1	0	1	0	0	1	1	0	1	1	6	M
Strock et al. (2023)	1	0	1	1	1	0	1	0	1	1	7	M
Papageorgiou et al. (2018)	1	1	0	1	0	1	0	1	1	1	7	M
Cook, Fourie & Crewther (2021)	1	1	1	1	0	1	1	1	1	1	9	H
Jurov, Keay & Rauter (2021)	1	1	1	0	1	1	0	1	0	1	7	M
Fensham et al. (2022)	1	1	1	0	1	1	1	1	1	1	9	H
Barrack et al. (2023)	1	1	1	0	0	1	1	0	1	1	7	M
Jurov et al. (2021b)	1	1	1	0	1	0	0	1	1	1	7	M
Kojima et al. (2020)	1	1	1	1	0	0	0	0	1	1	6	M
Kettunen et al. (2021)	1	1	1	0	0	1	0	1	0	1	6	M
Vardardottir et al. (2024)	1	1	1	0	0	1	1	0	1	1	7	M
McKay et al. (2022)	1	1	1	0	1	1	1	1	1	1	9	H

**Note:**

The quality assessment scale is a 1–10, with Low quality on a scale of 1 and High quality on a scale of 10. The scale division is 1–4 for low quality, 5–7 for Medium quality, and 8–10 for high quality. The quality assessment technique found that 26 research articles were high quality, and 13 medium quality items.

## Results and Discussion

The current review aims to investigate the following of athletes in competitive sports: the impact of cognitive functions on the motor performance of athletes, the role of reduced nutritional intake and LEA on the cognitive and motor abilities of the athletes and finally, to highlight the use of cognitive performance to use as first-level indicators of RED-S or FAT.

### Cognition and motor performance of athletes

Soccer-specific motor skills could be improved with appropriate cognitive interventions and sports training (Friebe et al., 2024). The cognitive intervention comprised agile multiple ball tracking tasks. Adding cognitive intervention with a decision-making component before soccer skill training seems to transfer the learning gains from the cognitive component to athletic movements (Scharfen & Memmert, 2021). The combination of cognitively challenging tasks, such as brain endurance tests and physical training, seems to increase the performance of athletes in competitive sports such as soccer (Staiano et al., 2022). The use of cognitive interventions remains advantageous and disadvantageous based on the position and duration of the cognitive intervention during skill training (Gantois et al., 2020). The prolonged incidence of cognitive tasks results in a tired state of the brain, compromising executive functions and the diminished ability to pay attention to task goals resulting in a mental state called mental fatigue (Marcora, Staiano & Manning, 2009).

### Cognitive decision-making component

Mental fatigue leads to impairment in decision-making and decreases the performance of athletes in competitive sports such as soccer (Huijgen et al., 2015; Romeas, Guldner & Faubert, 2016). Decision-making is an essential perceptual and cognitive skill required for elite performance in sports. Hence, coaches of elite athletes have incorporated the perceptual-cognitive component in their skill training (Starkes, Cullen & MacMahon, 2004). Therefore, elite soccer players trained with such components outperform novices in small-sided game tasks (Piggott et al., 2019). Small-sided games specifically emphasise the use of decision-making aspects to achieve the task goal (Davids et al., 2013). The decision-making component could be seen as increased brain activity in the regions (Lucia, Bianco & Di Russo, 2023).

### Nature of disinhibition

The capacity to make swift decision-making with appropriate motor action that could result in a quick response to an environmentally triggered stimulus is a quintessential feature of table tennis athletes (Castellar et al., 2019). To avoid unwanted responses during unexpected environmental conditions that could disrupt the outcome of the sport, the human brain uses an inhibitory mechanism to control the response (Criaud et al., 2016; Perri, 2020). The brain enters a preparatory state to provide a quick response before disinhibiting the inhibitory control providing proactive inhibitory control. The proactive inhibitory control might be one of the reasons for fast volitional reaction times in table tennis players when compared with non-athletes (Zhu et al., 2022).

### Neuroplasticity

The rapid disinhibition of the proactive inhibitory control might be caused by the fast transition between the inhibitory state and response state made possible by the connection that exists between the proactive inhibitory network and the primary motor cortex (Favre et al., 2013; Duque et al., 2017; Messel et al., 2019). Such rapid transition could be possible due to changes in the neural representations driven by experience, called experience-based neuroplasticity (Weisberg, van Turennout & Martin, 2007; Naito & Hirose, 2014). Hence, the rate of change of disinhibition in table tennis players were quicker when compared with non-athletes. Such rapid disinhibition could be due to neuroplasticity in the brain caused by substantial training in the sport (Jellinger, 2007; Raz & Lindenberger, 2013).

### Inhibitory and working memory neural circuits

The proactive inhibitory brain networks involved are the regions of the right inferior prefrontal gyrus, pre-supplementary motor area, right inferior prefrontal gyrus, striatum and subthalamic nucleus (Coxon, Stinear & Byblow, 2006; Meyer & Bucci, 2016). The event-related potential-electroencephalogram (ERP-EEG) acquired during Stroop tasks for elite table tennis players showed an interaction between cognitive functions and expertise of the sport (Huang et al., 2024). Substantial professional training for expert badminton players activated the left frontal-parietal area (left FPA), right dorsolateral prefrontal cortex (right DLPFC) and left ventrolateral prefrontal cortex (left VLPFC) during working memory tasks (Song, Xiang & Zhong, 2024).

### Sport-specific cognitive characteristics

The cognitive characteristics differ for interceptive, strategic and static sports (Yongtawee et al., 2022). The athletes exposed to higher demanding physical activity were shown to improve their higher cognitive functions-executive functions. The changes in the volume of grey matter in the cerebellum and basal ganglia seem to relate to the intensities of exercises performed, revealing the correlation between the activation of neural circuits and the intensity levels of the sport (Zhang et al., 2022). The higher blood serum BDNF, S100B, and neuron specific enolase (NSE) levels in high-intensity intermittent exercise (HIIE) than in the other groups showed improved cognitive performance with the Stroop test (Buzdagli et al., 2024). Table 2 shows the role of cognition abilities on the motor performance of athletes.

**Table 2:** Role of cognition abilities on motor performance of athletes.

Name	Population	Control/comparison group or condition	Task/Test/Experimental setup/Methods details	Results	Conclusion
Staiano et al. (2022)	*N* = 22 football players	Randomized to brain endurance training (BET) group or the control (CTL) group	4 weeks-40 physical training for BET & CLT. BET-cognitive training, CTL-neutral noises. 30–15 intermittent fitness test (IFT), soccer-specific reactive agility test (SSRAT), sprint ability random test (SART), and psychomotor vigilance (PV) and Stroop (SP) tasks/tests for all participants	Fast performance in BET than CTL for SART, SSRAT, IFT. Errors reduced in BET than CTL for SSRT Fast response in BET than CTL for SP. Lapses in BET than CTL for PV.	BET along with standard training is effective in improving the performance of the athletes
Friebe et al. (2024)	*N* = 42 adult male football players	Randomized to agility with multiple object tracking, change of direction (COD) and agility groups	Before and after 6-week training period using a dribbling test with and without a cognitive task (DTC and DTWC), Loughborough soccer passing test (LSPT), modified T-Test (TT) and random star run (with and without a ball) (RSR).	Improvement in performance after 6-weeks of training: TT, DTWC for all groups. DTC, RSR for agility groups. LSPT showed no significant improvements.	COD training focuses on pre planned changes of direction and dribbling, motor-cognitive agility therapies greatly improved agility and football specific transfer effects that require decision-making and multitasking.
Gantois et al. (2020)	*N* = 20 professional soccer male athletes	Randomized to CTL, 15 min SP, 30 min SP conditions	Decision-making analysis was conducted using the game performance assessment instrument (GPAI).	Reduction in passing decision-making performance for 30 min SP condition than 15 min SP and CTL.	Prolonged cognitive tasks influence male professional soccer players’ passing decision-making performance during a full-length training match.
Piggott et al. (2019)	*N*_1_ = 17 higher-skilled Western Australian Football League (WAFL) players.	Skilled WAFL and amateur players groups	WAFL and amateur players participated in three small-sided games lasting three minutes each.	According to a linear mixed model analysis, WAFL players outperformed amateur players in total score. WAFL players total score predicted the disposal efficiency.	Evaluation of disposals showed that higher-skilled players had significantly superior decision-making compared to lesser-skilled players.
	*N*_2_ = 23 lesser-skilled amateur players,				
Lucia, Bianco & Di Russo (2023)	*N* = 52 young semi-elite basketball players.	Randomized to the experimental (Exp) and CTL groups	EXP group participated in cognitive-motor dual-task training (CMDT). CTL group received standard training. Dribbling tests and a cognitive response task were performance evaluations. Brain activity was quantified using the P1, N1, and P3 components.	There was 13% improvement in sport-specific performance for Exp than Con group. Task speed and accuracy increased by 5.4% and 25.8%. EXP group showed increased brain activity.	CMDT received by EXP group increased brain activity for decision-making processes.
Zhu et al. (2022)	*N*_1_= 27 table tennis players.	EXP and CTL groups	The time of target presentation was adjusted to occur during different stages of the disinhibition process using a cue-target detection task with varied cue-target onset asynchrony (CTOA).	The disinhibition time was 200 ms for table tennis players when compared with 300 ms for non-athletes.	The neuroplasticity in the brain developed due to substantial practice of the sport could be a potential reasons for faster reaction times in table tennis players than in non-athletes
	*N*_2_ = 27 non-athletes				
Huang et al. (2024)	*N*_1_ = 20 table tennis players.	EXP and CTL groups	Coloured and spatial SP tasks were used in the study along with event related potential from EEG.	In the coloured SP task, athletes showed less Stroop effects on late sustained potential (LSP) parietal electrodes and less negative amplitude on the frontal electrodes than non-athletes. The athletes showed reduced reaction time amplitudes and Stroop effects on LSP for spatial SP task.	The findings emphasize the relationship between cognitive and table tennis expertise. These findings could help creation of training regimens to improve athletes’ cognitive skills, for competitive performance.
	*N*_2_ = 20 non-athletes				
Song, Xiang & Zhong (2024)	*N*_1_ = 22 athletes.	EXP and CTL groups	Behavioral performance and prefrontal cerebral oxygenation levels were assessed. Functional near infra-red spectroscopy (FNIRS) was used to quantify the blood oxygenation levels.	There were significant activations in left FPA, right DLPFC, and left VLPFC for badminton athletes than non-athletes.	Long-term professional training in badminton primarily activates the left FPA, right DLPFC, and left VLPFC during working memory tasks.
	*N*_2_ = 30 non-athletes				
Yongtawee et al. (2022)	*N* = 120	Three athletes and one non-athletes groups	Executive functions such as cognitive flexibility, inhibition with spatial ability and information processing were tested. Tests administered-mental rotation test, flanker test, simple reaction time, choice reaction time.	There was a significant difference in the cognitive functions across different sports types. Boxers showed advanced visuospatial functioning and processing speed. Soccer players showed better working memory and cognitive flexibility. Shooters showed faster visuo-perceptual processing than other groups.	Different cognitive characteristics for different sports enables selection of athletes based on cognitive abilities
	30 boxers, 30 shooters, 30 soccer players, 30 non-athletes.				
Koch & Krenn (2021)	*N* = 75 elite athletes	Closed skill and open skill groups	Executive functions (EF) were assessed with the following tests-flanker test, design fluency test, trail making test, and 2-back test.	The sports that required higher cognitive demands showed better scores for working memory and cognitive flexibility.	The cognitive demands provided by unpredictable environment of open-skill sports seems to play a vital role in development of athlete’s EF to higher levels for elite athletes participating in closed skilled sports.
Zhang et al. (2022)	*N*_1_ = 23 aerobic exercise experienced runners.	Aerobic and anaerobic groups	Magnetic resonance imaging (MRI) 3T with 32 channel head coil for brain scans.	MRI showed increase in the volume of gray matter in both cerebellum and temporal lobe for aerobic groups with increased activity in motor, parietal and frontal gyrus regions. The anaerobic group showed higher volume of gray matter in basal ganglia with increased activity in the posterior lobe of the cerebellum	Different brain activation modes respond to different levels of physical exercise, revealing neurological mechanisms that can distinguish athletes from different sports.
	*N*_2_ = 25 long time anaerobic sprinters				
Zhang et al. (2021)	*N*_1_ = 21 athletes.	EXP and CTL groups	Magnetic resonance imaging (MRI) 3T with 32 channel head coil for brain scans.	MR images showed significant increase in the volume of gray matter was seen in posterior lobe of cerebellum, frontal, temporal lobes and posterior cingulate, caudate and thalamus for elite athletes than non-athletes.	Anatomical & physiological plasticity of brain responsible for motor plan, motor action (execution) and supervision of motor action could be achieved after prolonged duration of motor skill training.
	*N*_2_ = 15 non-athletes				
Buzdagli et al. (2024)	*N* = included eight elite male boxers	Exercise and CTL conditions	Blood tests to identify BDNF, S100B, NSE) and cognitive ST were performed before and after the high intensity interval exercise (HIIE), moderate-intensity continuous exercise (MICE).	BDNF levels were considerably greater in HIIE than in CTL and MICE groups. S100B is significantly greater than in the CTL but no significant difference in the levels of S100B was found between HIIE and MICE groups. Levels of NSE were also significantly greater in HIIE than in control groups but NSE levels did not show significant change between HIIE and MICE groups.	HIIE is more effective than MICE at improving neuroprotection and cognitive performance. HIIE is preferred more than MICE, particularly in sports where cognitive performance is more crucial.

**Note:**

Cognitive intervention was beneficial or detrimental to motor task performance based on the intervention’s type, placement, and duration. Cognitive tests like Stroop tasks, further supported by EEG and FMRI studies, can show sport-specific improvements in cognitive abilities.

### Nutrition on cognition and motor performance of athletes

The nature of the sport often determines the type of movement patterns required to play the sport. Sports like soccer require players to perform a set of movement patterns such as long sprints, explosive jumps, and often changing the running direction (Bloomfield, Polman & O’Donoghue, 2007). Soccer players need eccentric contraction of muscles to produce game-required movement patterns (Jones, Bampouras & Marrin, 2009). Such eccentric contraction of muscles leads to delayed onset of muscular soreness and inflammation within 48 h after exercise (Armstrong, 1984; Fridén & Lieber, 2001). This muscle soreness can be caused by increased concentrations of creatine kinase in the blood (Nédélec et al., 2012; Russell et al., 2015). The supplement of n-3 polyunsaturated fatty acid (n3PUFA) with whey protein and carbohydrates reduces creatine kinase concentration and muscle soreness after an eccentric set of muscular concentration during soccer games (Philpott et al., 2018). Another practical nutritional strategy among athletes is ingesting fast absorption of caffeinated gum and mouth rinsing solution to increase brain neural activity and motor performance (Kizzi et al., 2016; Ehlert, Twiddy & Wilson, 2020).

### Caffeine intake and cognitive, motor performance

Female table tennis players showed increased speed and accuracy in their cognitive performance when taking the caffeinated gum and mouth rinsing solution compared with the control group (Pirmohammadi et al., 2023). The intervention group also improved service tests compared to the control group. Certain nutrition practices of using caffeinated gum and mouth rinsing solution seem to provide cognitive and physical motor level performance improvements, such as service accuracy and forehand drive for female table tennis players (Pirmohammadi et al., 2023). Caffeine intake in soccer athletes showed improvements in reaction time. One possible reason behind such performance could be the functioning of caffeine as an antagonist to the g-coupled-based adenosine receptors, causing a low threshold for motor unit recruitment, resulting in quick response action (Kalmar, 2005; Duvnjak-Zaknich et al., 2011). Improvements in decision time also might be because the caffeine intake would reduce the unwanted visual feedback during an athlete’s fatigue condition (Duvnjak-Zaknich et al., 2011). On the other hand, when the condition is not fatigued, the stimulatory neurotransmitters in the central nervous system cause improvements in agility time (Egesoy & Oksuzoglu, 2020). The caffeine intake in soccer players blocks adenosine receptors across the body to stimulate the CNS pathways and improve reactive agility performance (Egesoy & Oksuzoglu, 2020). Creatine nitrate and caffeine were ingested simultaneously in resistance-trained male athletes, and such intake was shown to improve cognitive performance in the Stroop test (Mabrey et al., 2024).

### Fatty acids supplements

The intake of w3 fatty acid (FA) and eicosapentaenoic acid (EPA) and docosahexaenoic acid (DHA) and docosapentaenoic acid (DPA) (levels increased in the blood) attenuates Nf-L levels in ASF players (Heileson et al., 2021). The reduction in Nf-L levels in the blood shows a decrease in inflammatory markers. The consumption of perilla oil rich in n-3 fatty acids in female volleyball players showed a reduction in constipation scores and levels of uremic toxin urinary indoxyl sulphate, highlighting the improvement of gut health in volleyball players with consumption of perilla oil (Kawamura, Nemoto & Sugita, 2023).

### Free radicals and oxidative stress

Exhaustive physical exercise practices with athletes lead to increased metabolism, thereby generating free radicals-highly reactive oxygen species (Rani et al., 2016). These reactive oxygen species tend to cause cell damage and also cell death of erythrocytes, leading to Fe toxicity in the body (Husain & Mahmood, 2017). Initially, the immune system responds to such causes. Still, the intensification of iron content in the blood during workouts might result in the degradation of the immune system with time, thereby leading to several upper respiratory syndromes (Valko, Morris & Cronin, 2005; Ward et al., 2011; Gleeson & Pyne, 2016; Walsh & Oliver, 2016). Antioxidants are used to neutralise the reactive oxygen species, protecting the cells from damage. Few natural supplements are believed to have biologically active compounds that will reduce oxidative stress on the body (Sikora, Broncel & Mikiciuk-Olasik, 2014).

### Natural antioxidant and iron profile

One such supplement is chokeberry, which is believed to contain compounds such as flavonoids, anthocyanin, and phenolic acids (Sueiro et al., 2006). These compounds tend to have a positive influence on the health of the athletes. The study examined the importance of chokeberry supplements before and after performing physical exercise on young football players. There was a significant change in the iron profile of the blood assessed using parameters such as albumin, myoglobins, hepcidin and ferritin. The prooxidants, such as thiobarbituric acid reactive substances and 8-oxo-2′-deoxyguanosine, did not significantly change with the supplement. Thereby showing the less potency of the chokeberry juice in improving the total antioxidant capacity of the body of athletes (Stankiewicz et al., 2021).

### Importance of the type of diet

A diet that contains proteins, phosphorus, potassium, magnesium and calcium, such as milk, cheese, meats, and eggs, increases the acidity of the food (Adeva & Souto, 2011). Such an increase in the acidity of the food could lead to potential renal acid load (PRAL) kidneys, causing several health issues such as chronic kidney disease, cardiovascular disease, bone disorders and reduction in muscle mass (Cordain et al., 2005). Hence, research has shown that using alkaline-based diets with induced alkaline supplements improves health and athletic performance (Deldicque & Francaux, 2008; McNaughton et al., 2016). The alkaline-based diet for 400 m sprint athletes improved their sprint performance (reduction in lapse time) compared to those with acid-based diets (Limmer, Eibl & Platen, 2018). Thereby highlighting the importance of an appropriate alkaline-based diet to improve athletic performance.

### Carbohydrate supplement

The sodium bicarbonate (SB) supplement with chronic and acute conditions tested on hockey players showed that chronic SB supplements increased the anaerobic capacity of the hockey players. In contrast, acute SB supplements increased the field performance during actual game tests (Durkalec-Michalski et al., 2020). Using carbohydrate beverages at regular intervals improves the skill performance and capacity to perform high-intensity exercises in sports such as soccer (Phillips et al., 2012; Kingsley et al., 2014). Hence, a 12% carbohydrate electrolyte solution at half-time increased the athletes’ performance-both passing speed and accuracy increased. There was also an increase in the athletes’ anaerobic endurance running capacity, providing them appropriate opportunities to excel in the sport (Rodriguez-Giustiniani et al., 2019). The intake of carbohydrate supplements-carbohydrate electrolyte, carbohydrate electrolyte protein solutions (CES and CEPS) showed improvement in the cognitive ability of male soccer players (Sun, Cooper & Chak-Fung Tse, 2020).

### Addition of proteins to supplements

The need for protein was always sidelined due to their non-support of anaerobic metabolism for fast contracting muscles (Ho et al., 2018). In recent research, protein has been seen as a supplement that could accelerate the recovery of fatigued fast, contracting muscles (Betts et al., 2007; Thomas, Morris & Stevenson, 2009; Lunn et al., 2012). A sport like basketball requires the recovery of tired muscles to accomplish the task goal. Adding protein to the carbohydrate supplement seems to increase the cerebral oxygenation in the frontal brain, improving the performance of high-intensity exercises in basketball players (Timinkul et al., 2008). Moreover, the protein with lesser carbohydrate supplement seemed to spike the insulin secretion with lesser glucose levels in the blood, resulting in quick recovery of the fatigued muscles *via* glycogen repletion (Ho et al., 2018). The adverse effects of dehydration (appropriate intake of fluids) showed a decline in elite Taekwondo athletes’ cognitive abilities-more dehydration also resulted in less cognitive functions (Zheng et al., 2024). Table 3 shows the role of nutrition on athletes’ cognitive and motor performance.

**Table 3:** Role of nutrition on athletes’ cognitive and motor performance.

Name	Population	Control/comparison group or condition	Task/Test/Experimental setup/Methods details	Results	Conclusion
Zheng et al. (2024)	*N* = 12 Taekwondo (TKD) athletes	A randomized cross over design with three different levels of hydration was used.	The cognitive abilities were measured using Vienna test system (speed reaction time test).	The group with no restriction on consumption of fluid showed higher success rate in the front kick (speed reaction time test) when compared to the groups that had restricted fluid access.	TKD athletes showed negative effects of hydration on cognitive skills.
Philpott et al. (2018)	*N* = 30 young competitive male soccer players	Categorized into three groups: a fish oil, protein and carbohydrate (FO, PRO and CHO)	Throughout the 6-week supplementation period, the participants were instructed to continue exercise training as well as habitual diet routine.	After 6 weeks, 3 PUFA/total PUFA composition increased in the blood by 58% in the FO group and experienced less leg soreness than other groups. The LSPT did not reveal any appreciable changes from baseline over the 72-h recovery period.	n-3PUFA along with leucine, whey protein, and carbohydrate intake reduces muscle soreness and creatine kinase levels in competitive soccer players after eccentric activity.
Pirmohammadi et al. (2023)	*N* = 18 female table tennis players.	Crossover, double-blind, randomized design and were randomized into one of three conditions consuming caffeinated gum (CG), mouth rinsing after drinking coffee (CMR), or taking placebo capsules (PLA).	A 1-week washout period was used between conditions. Functional, skill, and cognitive tests were measured at each session.	In comparison to the placebo (PLA), both caffeinated gum (CG) and coffee mouth rinsing (CMR) greatly increased agility and decreased errors in cognitive tests. While CMR enhanced isometric hand strength, forehand drive, service accuracy, CG boosted hand movement speed.	Sources of caffeine with early absorption (CMR and CG) are effective ways to raise the performance. On functional and cognitive assessments CMR and CG are having a greater impact than on skill tests.
Kawamura, Nemoto & Sugita (2023)	*N* = 36 female athletes	Six times per week training. The participants were assigned to three groups depending on perilla oil intake: high, low oil and placebo (HOI, LOI and PLA).	For 8 weeks, HOI and LOI groups consumed jelly with perilla oil-, while the PLA group received jelly without perilla oil. The gut microbiota, constipation scores, and urine biochemical indicators were assessed before and after the intervention.	Proteobacteria decreased in HOI group, while beneficial Lachnospiraceae increased. Urinary indoxyl sulphate levels also decreased in HOI group. Constipation scores improved in HOI and LOI groups.	Daily intake of n-3 fatty acids from perilla oil can benefit trained female athletes by improving gut microbiota growth and function, and providing a valuable fuel source.
Stankiewicz et al. (2021)	*N*_1_ = 12 of young football players	Exp and CTL groups.	Chokeberry juice of 200 ml was given every day for 7 weeks. Participants completed a beep test before and after the supplementation period. Venous blood samples were collected for serum analysis before, after (immediately, 3 and 24 h) the beep test.	Serum levels of thiobarbituric acid reactive products, 8-hydroxy-2-deoxyguanosine, total antioxidant capacity, iron, hepcidin, ferritin, myoglobin, and albumin, and morphological blood parameters were determined.	Chokeberry juice supplementation had no effect, indicating that the juice lacked significant antioxidant capacity.
	*N*_2_ = 8 of young football players				
Limmer, Eibl & Platen (2018)	*N* = 11 athletes	Randomized, single-blind, counterbalanced crossover trial design with Regular diet and either a 4-day alkalizing (BASE) or acidifying (ACID) diet conditions.	The trials, held 1 week apart, consisted of 400-m runs on a tartan track in a random order.	400-m performance speeds were considerably faster in the BASE condition than in the ACID trial. Furthermore, blood lactate levels and urinary pH were considerably higher after the BASE diet, indicating increased blood or muscle buffer capacity under the alkalizing diet.	The findings imply that ingesting natural, low-PRAL alkalizing foods and beverages can improve performance without the need of dietary supplements such as NaHCO_3_ or sodium citrate, giving athletes a natural and option to improve sprint performance.
Rodriguez-Giustiniani et al. (2019)	*N* = 15 soccer players	Double-blind randomized cross-over design with carbohydrate-electrolyte (CHO), placebo-electrolyte (PL), or water (Wat) beverages conditions.	Blood samples were taken before each half-hour and every 15 min while exercising. Physical performance (sprinting, jumping, distance, acceleration/deceleration), technical performance (dribbling), and cognitive performance were all evaluated, as well as ratings of perceived effort (RPE) and stomach discomfort.	The CHO drink increased mean accelerations (>1.5 m/s^2^), dribbling speed after 60 min, and sprinting speed during self-paced exercise. Blood glucose levels in the CHO group dropped by 27% after 60-min. RPE were similar across all trials. Cognition declined post-exercise and was not improved by CHO intake.	The last 30 min of exercise were markedly improved by consuming a 12% CHO beverage compared to water and placebo. Post-exercise cognitive decline was not prevented by it.
Ho et al. (2018)	*N* = 15 basketball players	Randomized, counterbalanced crossover study with consumption of carbohydrate based either a high-protein or isocaloric low-protein control supplement (6.25 kcal/kg) conditions.	Cycling challenge at 80% VO_2_max. Throughout both sessions, near-infrared spectroscopy were used continuously to monitor oxygen saturation and blood perfusion (total hemoglobin) in the frontal brain.	The group with high-protein supplement showed elevated insulin and lower glucose levels throughout recovery than the low-protein experiment along with increased cerebral oxygen saturation, lowered cerebral blood perfusion during exercise, further prolonging the riding time by 16% than low-protein supplement group.	Consuming a high-protein supplement improves fatigue recovery by increasing cerebral oxygenation during exercise, reducing the brain’s need for peripheral blood supply.
Egesoy & Oksuzoglu (2020)	*N* = 48 healthy male youth soccer players	Randomized, counter-balanced, single-blind, and repeated-measures experimental design participants placed in to caffeine, decaffeinated or placebo, no coffee or baseline groups.	Caffeine group consumed either 6 mg·kg^−1^ of caffeinated coffee, placebo group consumed 6 mg·kg^−1^ of decaffeinated coffee, and baseline group consumed no coffee at all. The researchers examined movement, sprint, total agility and decision time (MT, ST, TAT and DT).	Caffeine ingestion significantly improved MT compared to the placebo group. Additionally, ST showed significant difference in baseline condition than in the caffeine and placebo groups. Caffeine group showed significant improvements in TAT and DT when compared to baseline and placebo conditions.	Caffeine intake improves ST, TAT, and DT components as compared to the baseline and placebo. Caffeine ingestion improves soccer players’ reaction agility performance.
Durkalec-Michalski et al. (2020)	*N* = 24 trained male field hockey players	Randomized, placebo-controlled, crossover trial with chronic and acute supplementation conditions	Progressive-chronic protocol with incremental doses ranging from 0.05 to 0.2 g/kg, and an acute one-off dose of 0.2 g/kg. Participants completed a discipline-specific field performance test, followed by two Wingate anaerobic tests (WAnTs), before and after each supplemental session.	Progressive-chronic SB supplementation increased anaerobic capacity during the first bout of WAnTs, with improvements in mean power, peak power, and power carry threshold. Acute SB supplementation did not increase anaerobic capacity, but improved performance times in field-specific tests than a placebo or no treatment.	The performance of field hockey athletes is enhanced by SB supplementation, regardless of whether it is administered in acute or chronic doses, according to a randomized controlled trial.
Mabrey et al. (2024)	*N* = 12 male athletes	Double-blind, randomized crossover trial with creatine nitrate, caffeine and both as conditions.	7 days of creatine nitrate (5g per day), caffeine (400 mg per day) and both. Cognitive function test along with cardiovascular test were performed after ingestion of the supplement. Pre-test-standard resistant exercises were performed. Blood samples were taken in specific intervals.	After creatine nitrate and caffeine ingested simultaneously showed significant improvement in SP color test when compared to the separate ingestion. The co-ingestion did not result in any side-effects other than showing improvements with the cognitive performance.	The improvement with the cognitive function without negative side effects up to 7 days could be caused due to the ingestion of caffeine with creatine nitrate.
Heileson et al. (2021)	*N*_1_ = 31 American style football (ASF) athletes	EXP and CTL	w-3 fatty acid (w3-FA) formulation supplement with the composition of the supplement docosahexaenoic acid (DHA)—2,000 mg, Eicosapentaenoic acid (EPA)-560 mg, Docosapentaenoic acid (DPA)-320 mg was given. The supplement was given during the preseason as well as throughout the season. The control ASF athletes *N* = 35 did not receive the supplement. Blood samples were taken in specific intervals throughout the study.	ASF treatment group showed increase in the EPA & DHA leading to increase in the O3I (Omega 3 Index) than CTL. Treatment group showed the decrease in the neurofilament light (Nf-L) levels. As the control group that did not take w3-FA supplement, showed elevated values of Nf-L compared to treatment group.	Supplementation with EPA, DPA, and DHA w3-FA shown to have cardio- and neuroprotective effects in ASF athletes.
	*N*_2_ = 35 American style football (ASF) athletes				
Sun, Cooper & Chak-Fung Tse (2020)	*N* = 16 male soccer players.	Randomized cross-over design with carbohydrate electrolyte, carbohydrate electrolyte protein solutions (CES and CEPS) and placebo conditions.	Cognitive test were performed-Loughborough intermittent shuttle test (LIST), rapid visual information processing test (RVIPT) and visual search test (VST).	The accuracy of RVIPT after LIST and response time in VST improved for CES and CEPS trials compared to placebo trials. Also, CEPS consumption showed to improve accuracy in VST than the consumption of CES.	The CES and CEPS consumption groups showed improvements in cognitive performance (sustained vigilance and attention) of male college soccer players during soccer-specific exercise protocols.
Zhu et al. (2020)	*N* = 14 male soccer athletes	Single-blinded, randomized, cross-over experiment with Carbohydrate electrolyte solution (CHO), CHO and mindfulness (CHOM) audio intervention and non-carbohydrate electrolyte solution (control group) conditions	Cognitive test were performed-SP test, Corsi block tapping test (CBT) and RVIPT.	The performance in the SP task for CHOM group was better than control, CHO group. CBT performance was faster for control & CHOM groups for pre and post-test comparisons. In RVIPT, less time was spent in finding missing number by CHO group compared with other groups	Exposed preliminary evidence of the favorable effect of mindfulness based intervention combined with CHO intake on athletes’ cognitive function, as well as both positive and negative effects of CHO consumption.
Goulart et al. (2023)	*N* = 119 esports athletes	Repeated measures ANOVA with food intake groups	Cognitive test was performed with 3D multiple objects tracking test (3DMOT) with Neurotracker software (NTx)	NTx was significantly different for those who took appropriate amount of protein than those who did not take appropriate amounts of proteins	Protein and specific micronutrients contribute to increased cognitive performance in esports participants.

**Note:**

The intake of appropriate nutrient supplements, such as n-3 PUFA, carbohydrates, bicarbonate, protein, electrolytes, water hydration, and an alkaline diet, seems to influence the cognitive and motor abilities of athletes substantially.

### Energy availability on cognition, motor performance & health of athletes

Female athletes involved in competitive sports tend to develop female athlete triad syndrome (FAT) (now commonly called as RED-S) due to a lack of energy to serve the needs of the sports they participate (De Souza et al., 2014; Mountjoy et al., 2014). Once the dietary intake fails to meet the energy requirements to perform sports, it leads to complications in female athletes, such as menstrual disturbances, bone metabolism, LEA or deficiency (De Souza et al., 2014). Less dietary intake could be caused by an eating attitude of conscious restriction of energy intake called cognitive restrain or fear of becoming fat called drive for thinness. Such attitude toward eating could probably lead to female athlete triad syndrome (Strock, De Souza & Williams, 2020; Souza et al., 2021). Athletes with female athlete triad syndrome experience menstrual disturbances such as oligomenorrhea (oligo: irregular menses) and amenorrhea (amen: no menses) (De Souza et al., 2010). A 12-month change in the dietary plan for the exercising female population showed that a gradual increase in calorie intake reduces the menstrual disturbances in oligo and amen individuals (Strock et al., 2023).

### LEA and bone metabolism

The dietary restrictions and exercise requirements in specific sports cause LEA in female athletes, resulting in FAT (Loucks, Kiens & Wright, 2011). LEA reduces bone mass, deformities at the bone’s microarchitecture and a high risk of bone fracture (De Souza et al., 2008; Barrack et al., 2014; Southmayd et al., 2017). The exercising females with no menstrual disturbances (eumenorrheic: regular) were also vulnerable to adverse changes in bone metabolism. Eumenorrheic exercising females were subjected to energy restriction through less dietary intake and exercise regimes. The LEA caused by the restriction of diet showed a significant decrease in bone formation indicated by bone turnover biomarker P1NP, Amino-terminal propeptide of procollagen type-1 with no effect on bone resorption indicators (no change in the concentration β-CTX: C-terminal cross-linked telopeptide of type I collagen) (Song, 2017; Papageorgiou et al., 2018). The LEA caused by exercise showed no significant change in bone formation and bone resorption indicated by bone turnover biomarker P1NP, Amino-terminal propeptide of procollagen type 1 and β-CTX: C-terminal cross-linked telopeptide of type I collagen (Song, 2017; Papageorgiou et al., 2018).

### Bone health and carbohydrate restriction

Elite race walk athletes were induced LEA, and bone metabolism was assessed (Mountjoy et al., 2014). The P1NP bone formation markers were reduced at rest and exercise conditions after the restriction of intake of carbohydrates (Wu et al., 2021; Fensham et al., 2022). At the same time, the exercise-related effects were seen in the bone resorption marker β-CTX. During the LEA, the P1NP remains unchanged while the β-CTX increases (Wu et al., 2021; Fensham et al., 2022). Hence, the restriction of carbohydrates for a short period of time reduces bone metabolism in male athletes for exercise and rest conditions (Fensham et al., 2022). The female athlete triad has a significant role in determining female athletes’ bone health and metabolism (Papageorgiou et al., 2017). The female adolescent athletes with clinical markers for LEA showed lower mineral density across the body and lumbar support (Soltani et al., 2016; Barrack et al., 2023).

### Menstrual and hormone levels

The hypothalamic-pituitary-gonadal (HPG) & hypothalamic-pituitary-adrenal axis (HPA) control fertility and stress in eumenorrheic women (Kudielka, Hellhammer & Wüst, 2009; Andreano et al., 2018). In eumenorrheic women, testosterone levels change with exercise levels across the menstrual cycle phases (Cook, Kilduff & Crewther, 2018). The elite female athletes, mostly induced with both physical and cognitive stresses, showed changes in the concentrations of testosterone and cortisol (Hlavacova et al., 2008; Childs, Dlugos & De Wit, 2010; Cook, Kilduff & Crewther, 2018; Crewther & Cook, 2018). Further, the concentration of testosterone and cortisol reactivity correlated with the baseline testosterone concentration, highlighting the role of the complex interplay between HPA and HPG axis (Cook, Fourie & Crewther, 2021).

### Cognitive restraint and LEA

The LEA in endurance athletes showed a reduction in performance (Jurov et al., 2021b). The LEA reduced the vertical jump height in endurance athletes (Jurov et al., 2021b). These athletes also showed higher cognitive restraint with LEA, and cognitive restraint was also an early marker of energy conservation (Jurov et al., 2021a). Endurance performance was also affected for the male athletes with LEA (Jurov, Keay & Rauter, 2021). LEA correlated with testosterone levels, indicating the deterioration of performance followed by eating disorders in healthy male athletes (Jurov et al., 2021a; Jurov, Keay & Rauter, 2021). The consecutive training of endurance runners showed less glycogen in the muscles under LEA conditions (Kojima et al., 2020). During the training camps, the cross-country skiers showed difficulties reaching their EA and carbohydrate requirements, resulting in LEA and RED-S (Kettunen et al., 2021). Female athletes showed negative implications for their health with repeated exposure to LEA and low carbohydrate (LCHO) intake (Vardardottir et al., 2024). Appropriate EA and CHO could prevent RED-S. The athletes’ Low carbohydrate/high fat with high energy availability (LCHF) diet and CHO intake restriction cause unfavourable immune and iron stress responses to exercise (McKay et al., 2022). Table 4 shows the role of energy availability on cognition, motor performance & health of athletes.

**Table 4:** Role of energy availability on cognition, motor performance & health of athletes.

Name	Population	Control/comparison group or condition	Task/Test/Experimental setup details/Methods	Results	Conclusion
Strock et al. (2023)	*N*_1_ = 40 Oligo/Amen and Cal women,	Randomized control trial with Oligo/Amen and Cal women, Oligo/Amen Control women, and (OVref) (baseline only) groups	Body composition was assessed *via* dual energy x-ray absorptiometry once per month for the first 3 months then once every 3 months. Nutritionist and Psychologist Interviews, Questionnaires were conducted every 2 weeks for the first 3 months then monthly for the rest of study.	Increased energy intake led to higher fat mass percentages but had no impact on psychological and behavioural outcomes in the Oligo/Amen and Cal group than Oligo/Amen control group. There was decrease in cognitive restraint and improvement in resilient coping among all groups over a 12 month duration.	A long-term dietary intervention in women with menstrual abnormalities improved body weight and fat mass without increasing disordered eating attitudes, psychological stress, or depressive symptoms in Oligo/Amenorrhea who exercise.
	*N*_2_ = 36 Oligo/Amen control women				
	*N*_3_ = 37 Ovulatory women (OVref) (baseline only)				
Papageorgiou et al. (2018)	*N* = ten eumenorrheic women	Random cross over design with 3-day conditions in random order: controlled energy availability (CON), LEA *via* dietary restriction (D-RES), and LEA by increasing exercise expenditure (E-RES).	Blood samples were collected at baseline and after the 3-day period to measure markers of bone turnover (β-CTX, P1NP), calcium metabolism (PTH, Ca, Mg, PO4), and various hormones (IGF-1, T3, insulin, leptin, and 17β-oestradiol).	While bone resorption remained unchanged, D-RES significantly reduced bone production. R-RES had no discernible effect on bone metabolism. Hormone concentrations changed significantly and similarly at both LEA conditions.	Low EA from dietary energy restriction significantly decreased bone formation without affecting bone resorption. In contrast, LEA caused from energy expenditure after exercise did not impact the bone metabolism.
Cook, Fourie & Crewther (2021)	*N* = 30 athletic women	Semi randomized design with hormonal baseline, change of scores measures, and stressor type conditions.	Physical (4 × 6-s bike sprints) and psychological (5 × 2-min cognitive assessments with social evaluation) stressor on days 7 (D7), 14 (D14), and 21 (D21) of menstrual cycle. Salivary testosterone and cortisol levels were assessed both at baseline and during acute alterations.	The study found that the testosterone response to stressors was stronger on D14, but cortisol levels were less responsive compared to D7 and D21. Baseline testosterone, which varied significantly between persons and peaked on D14, was found to be linked with testosterone and cortisol responsiveness to stressors on a between-person basis.	Baseline testosterone levels correlate with testosterone and cortisol responses to physical and psychological stressors. Endogenous testosterone influences hormone responses to stress, as demonstrated by individual variances and temporal fluctuations in naturally cycling athletic women.
Jurov, Keay & Rauter (2021)	*N* = 12 athletes	Cross-sectional controlled design with baseline stage and different stages of practice during LEA conditions	Energy availability (EA), monitored exercise energy expenditure (EEE) during training, and resting energy expenditure (REE) were assessed, and three performance tests (endurance, explosive power, and agility) were performed, as well as psychological questionnaires to assess baseline performance.	Reducing EA by raising EEE by 50% over 14 days resulted in significant reductions in body fat, testosterone, and hemoglobin, as well as declines in endurance and explosive power. This intervention showed no changes in REE. Psychological evaluations successfully identified the decrease in EA.	Male athletes lose endurance and explosive power before any harmful health effects occur. Significant reductions in EA could lead to poor eating behaviors, and the psychological questionnaires used were more sensitive to EA changes than blood markers.
Fensham et al. (2022)	*N* = 28 top racewalkers	Parallel group design with baseline, high-carbohydrate, high-energy diet (CON) control, low carbohydrate/high fat/high energy availability (LCHF), LEA groups.	Participants completed a 25-km racewalk following each phase, with blood samples collected while fasting, pre-exercise, and at 0, 1, and 3 h post-exercise to measure markers such as osteocalcin (gla-OC and glu-OC), carboxyterminal telopeptide (CTX), N-terminal peptide (P1NP), and procollagen-1.	Following adaptation, LCHF showed lower fasted P1NP, gla-OC, and glu-OC, which were all substantially different from CON. Both LCHF and LEA had significantly greater CTX levels from pre-exercise to 3 h post-exercise, however only LCHF had lower P1NP levels.	There was reduction in bone production markers during exercise after restriction of carbohydrate for short period in time, but there was increase in bone resorption markers after exercise. In LEA, bone production markers are maintained during exercise while resorption rises. Adequate energy and carbohydrate intake can help elite endurance athletes avoid the detrimental effects of exercise on bone turnover.
Barrack et al. (2023)	*N* = 209 athletes	Cross sectional design with athletes having/not having clinical indicators of LEA conditions.	Bone mineral density and body composition assessed using dual-energy x-ray absorptiometry. Athletes with primary or secondary amenorrhea or clinically underweight were considered to have limited energy availability. Questionnaire was used to evaluate disordered eating behaviour.	80% of the athletes did not fulfil the criteria for disordered eating or stated a wish to lose weight. Furthermore, athletes with clinical symptoms of limited energy availability had lower Z-scores for lumbar spine and whole body bone mineral density than those without such indicators	Many female adolescent athletes with LEA did not deliberately restrict their diet, implying that unexpected causes contribute to LEA and emphasizing the need for improved nutrition education for this group.
Jurov et al. (2021b)	*N* = 12 elite endurance athletes	Intervention cross-sectional controlled design with baseline stage and different stages of practice conditions during LEA condition.	Blood samples were collected regularly. The psychological assessment questionnaires were filled by participants to assess the cognitive restrain during lower energy availability.	The reduced energy availability (22.4 ± 6.3 kcal/kg FFM/day) significant low hemoglobin content in the blood due to lower iron and IGF-1. Explosive power of the lower extremities were reduced and the lactate mechanism seemed to change. Cognitive restraint was high for LEA scenario	Men endurance athletes may have a lower threshold for LEA than females. It also demonstrated that cognitive restraint can assist in detecting early signals of conservation of energy. The significant cognitive restraint seen in the sample emphasizes the importance of evaluating endurance athletes’ eating practices to prevent the possibility of disordered eating patterns.
Kojima et al. (2020)	*N* = Seven male long-distance runners	Randomized crossover design LEA and normal energy availability (NEA) conditions	Endurance training exercise performed under LEA (18.9 ± 1.9 kcal·kg FFM·d) and NEA (52.9 ± 5.0 kcal·kg FFM·d) for 3 days. Muscle glycogen content was analyzed from blood and urine samples collected during the course of the training.	The body weight, fat mass, muscle volume and glycogen reduced significantly in LEA than in NEA.	3 days of endurance training with LEA lowered muscle glycogen content and body weight, but had no meaningful effect on endurance capability.
Kettunen et al. (2021)	*N* = 19 female cross-country skiers	Cross sectional observation design with before (PRE) and after (POST) training camp conditions	Fasting blood analysis was performed PRE and POST the camp. The metrics such as blood lactate (LA), heart rate and rating of perceived exertion (RPE) were collected in the following tests-submaximal treadmill running test, counter movement jump (CMJ) and reactive jump test (RJ).	Mean EA was 40.3 kcal/kg FFM/day. 58% of the participants showed sub-optimal EA. 37% of the participants had low EA. hemoglobin, leptin, T3, and insulin decreased values when compared between PRE and POST conditions. Whereas RPE and glucose values increased when compared between PRE and POST conditions. EA correlated with LA during the training period.	Many athletes struggled to achieve their energy and carbohydrate requirements during a training camp. Furthermore, keeping adequate EA is critical to avoiding overreaching and preserving performance during hard training periods.
Vardardottir et al. (2024)	*N* = 41 female athletes	Cross-sectional design with groups based on EA and CHO criteria. The groupings are as follows 1. Sufficient to optimal EA and Sufficient to optimal CHO intake (SEA and SCHO) 2. SEA and Low CHO intake (LCHO) 3.LEA and SCHO 4. LEA and LCHO	Body composition was measured using x-ray absorptiometry. Behavioural risk factors and self-reporting symptoms of RED-S was assessed using females, muscle dysmorphic, eating disorder questionnaires.	36.6% of participants categorized into the SEA and SCHO group. Recovery by the LEA group was worse when compared to that of the SEA and SCHO group.	Female athletes who are repeatedly exposed to LEA and LCHO consumption are more likely to experience a variety of unfavourable outcomes. Adequate EA and carbohydrate consumption are critical nutritional measures for avoiding RED-S.
McKay et al. (2022)	*N* = 28 elite racer walkers	The athletes were placed in either of the three diet groups. High CHO/EA group (CON), Low CHO High Fat (LCHF) and LEA.	25 km walk protocol was assigned to the athletes at the end of each phase. Glucose, cortisol and white blood cells were collected before and after the walk.	There was an increase in the white blood cell counts, cortisol levels post exercise for the LCHF group. There was decrease in the blood glucose levels lost from the training with the LCHF group with no changes in glucose levels for CON and LEA group. There was no significant change between LEA and CON groups for the biological variables measured from blood.	Short-term adherence to an LCHF diet had a detrimental influence on iron levels, immunological function, and stress responses, but no significant health impacts were observed in athletes with LEA compared to those with adequate energy availability. Athletes on short-term LEA should have enough carbohydrates and protein to maintain immunological health.

**Note:**

Lower energy availability in athletes caused health-related issues from irregular menstrual cycles, fertility axis, and bone metabolism. Cognition played a crucial part before low energy availability in the form of cognitive restraint and as an essential early marker after low energy availability in the form of changes in performance for the athletes.

### Summary

In summary, the cognitive skills of the athletes in competitive sports could be assessed using various tasks such as Stroop, tracking task memory tasks, *etc*. Such tests were used to train athletes appropriately to improve motor performance. The improvement in motor performance could result in the accomplishment of the task goal of the sport. The cognitive intervention should be selected in a specific phase of the skill training to avoid mental fatigue and loss of decision-making skills.

### Cognitive skills for motor performance

Decision-making skills are quintessential in competitive sports. Training involving interventional games with decision-making requirements was shown to improve the performance of the sport. Such decision-making cognitive skill training increased neural activity at the brain level. The different cognitive skills showed activity in the various areas of the brain based on the needs of the sports. One such area in the brain is the proactive inhibitory control to restrict unwanted movements. With substantial practice, inhibitory control would quickly disinhibit to give response for athletes when compared with novices. Such quick responses in competitive sports could be possible with experience-driven neuroplasticity, which could be identified with ERP & EEG analysis.

### Nutritional supplements for cognitive skills

Similar to specific cognitive skills required for particular sports, specific motor or movement patterns are necessary for their sports execution. These movement patterns in competitive sports should be eccentric and high-intensity, causing inflammation and muscle soreness. n3PUFA with whey protein and carbohydrate supplement reduces soreness and constipation and improves gut health. Caffeinated supplements tend to improve cognitive and motor performance by improving decision-making skills by increasing attention *via* the visual system and specific muscle group recruitment for reactive agile movements. The exhaustive physical demands of sports cause free radicals in the blood; hence, athletes use antioxidants to remove such reactive species. Therefore, Antioxidant consumption should be selected appropriately to avoid immune and upper respiratory disease in athletes.

### Type of diet and athletes’ performance

Further use of an acidic diet increases PRAL, thereby reducing athletic performance and resulting in health issues for athletes like cardiovascular disease and renal disorders. An alkaline diet with sodium carbonate supplements was preferred to overcome this issue, increasing athletes’ sprint performance. The carbohydrate and protein supplements improved endurance capacity and recovery after high-intensity exercise in competitive sports. Finally, negative hydration reduced cognitive performance in athletes; the decline was evident with reduced levels of BDNF, NSE, and S100B in blood serum. The changes in biomarkers speculate the possible role of nutrition that might result in the changes in neural representations for neural plasticity.

### Energy deficiency and cognitive restraint

Female athlete triad is developed in female athletes due to lack of appropriate energy intake. Similar to female athletes, male athletes have severe complications due to energy deficiencies. Hence, the syndrome was collectively termed relative energy deficiency in sports syndrome (RED-S) for both athletes. Female athlete triad led to complications such as bone metabolism, menstrual disturbances, and eating disorders due to voluntary restriction of diet. Female athlete triad could lead to menstrual disturbances, but appropriately long nutrition intervention could change the effect of menstrual disturbances in athletes. Another severe health concern in female athletes is a reduction in mass and microarchitecture of bone due to energy deficiency. The energy deficiency induced by the dietary intake showed a decrease in bone formation. The energy deficiency caused by the high-intensity energy intervention caused no significant reduction in bone formation and resorption in Eumenorrheic exercising females. The changes in bone metabolism were also seen in male athletes who were induced to have energy deficiency with carbohydrate intake restriction. In female athletes, the correlative interplay between testosterone and cortisol hormones is caused by cognitive and physical stresses. The LEA in male endurance athletes reduced athletic performance. The low energy reduced male athletes’ high-capacity vertical jumps and cognitive performance. Cognitive restraint was one of the first signs of energy conservation; the deterioration of performance and poor eating habits resulted from LEA in athletes.

### Cognitive test as assessment tool for RED-S/FAT

Hence, appropriate nutrition and dietary plans play a crucial role as a fulcrum to avoid relative energy deficiency and its consequences. Cognitive performance, vital for accomplishing goals in competitive sports, is positively and negatively dependent on nutrition and energy availability. Studies have shown a reduction in cognitive performance during induced energy deficiency experiments. On the other hand, appropriate nutritional supplements could either increase or decrease cognitive performance based on the time and position of the intervention. The changes in the neuronal activity in different brain areas also suggest a strong relation between cognitive performance and nutritional & energy deficits. Further changes in the biomarkers of experience-related neuroplasticity in the brain differentiate the athletes from the normal population. Hence, evaluation of cognitive performance with appropriate cognitive tests (Stroop, memory, ball tracking, *etc*.) could enable the coaches or athletes to assess the energy and nutritional deficits to prevent RED-S or FAT in competitive sports.

### Limitations and future direction

The current review articles showed a significant dependence of cognitive performance on nutritional and energy deficits. Even though several experimental data are available to study the cognitive, nutritional and energy deficiency in athletes, no machine learning (artificial intelligence) model is designed to predict athletes’ physical and mental abilities before the onset of RED-S. Designing a machine learning model incorporating cognitive, nutritional, and energy deficit data would improve the monitoring of athletes by predicting their physical and mental abilities. This model can use existing experimental data to identify correlations in the three major areas discussed. Once the machine learning model is generated, it could be integrated into mobile applications where athletes could play cognitive test-based games, permitting them to assess their vulnerability towards RED-S. This development can assist real-time monitoring and proactive intervention schemes, finally improving athlete health and performance.

## Conclusion

The review articles address the roles of cognition, nutrition and energy availability/deficiency in the performance of the athletes in competitive sports. It was identified that specific nutritional and dietary plans could prevent the onset of symptoms of RED-S and FAT in male and female athletes. Cognitive abilities seem to depend on the athletes’ nutritional and energy availability requirements. Athletes’ cognitive abilities were assessed based on their performance using appropriate cognitive tests. Hence, the current review suggests that cognitive performance could be used as an early and first evaluation measure to identify male and female athletes’ nutritional and energy deficiencies to combat RED-S and FAT.

## Additional Information and Declarations

### Data Availability

The following information was supplied regarding data availability:

This is a literature review.
